# From an understanding of etiopathogenesis to novel therapies—what is new in the treatment of celiac disease?

**DOI:** 10.3389/fphar.2024.1378172

**Published:** 2024-04-18

**Authors:** Kinga Skoracka, Szymon Hryhorowicz, Francesco Tovoli, Alberto Raiteri, Anna Maria Rychter, Ryszard Słomski, Agnieszka Dobrowolska, Alessandro Granito, Iwona Krela-Kaźmierczak

**Affiliations:** ^1^ Department of Gastroenterology, Dietetics and Internal Diseases, Poznan University of Medical Sciences, Poznan, Poland; ^2^ Doctoral School, Poznan University of Medical Sciences, Poznan, Poland; ^3^ Institute of Human Genetics, Polish Academy of Sciences, Poznan, Poland; ^4^ Division of Internal Medicine, Hepatobiliary and Immunoallergic Diseases, IRCCS Azienda Ospedaliero-Universitaria di Bologna, Bologna, Italy; ^5^ Laboratory of Nutrigenetics, Department of Gastroenterology, Dietetics and Internal Diseases, Poznan University of Medical Sciences, Poznan, Poland

**Keywords:** celiac disease, autoimmunity, HLA, novel therapies, gluten-free diet

## Abstract

Celiac disease, a chronic autoimmune disorder caused by genetic factors and exposure to gluten, is increasingly being recognized and diagnosed in both children and adults. Scientists have been searching for a cure for this disease for many years, but despite the impressive development of knowledge in this field, a gluten-free diet remains the only recommended therapy for all patients. At the same time, the increasing diagnosis of celiac disease in adults, which was considered a childhood disease in the 20th century, has opened a discussion on the etiopathology of the disease, which is proven to be very complex and involves genetic, immunological, nutritional, environmental and gut microbiota-related factors. In this review, we extensively discuss these factors and summarize the knowledge of the proposed state-of-the-art treatments for celiac disease to address the question of whether a better understanding of the etiopathogenesis of celiac disease has opened new directions for therapy.

## 1 Introduction

Celiac disease (CD) is a chronic, systemic small intestinal enteropathy that develops in genetically predisposed individuals. In human leukocyte antigen (HLA)-DQ2 and/or HLA-DQ8 positive people, exposure to dietary gluten activates immune response characterized by specific serum autoantibody response in IgA and IgG class—anti-transglutaminase IgA and anti-endomysial antibodies IgA and deamidated gliadin-related peptide IgA and IgG—which results in pathological changes in small intestine such as crypt hyperplasia, lymphocyte infiltration, and villous atrophy (Ludvigsson and Murray, 2019a; Pinto-Sanchez et al., 2021).

The global prevalence of CD is estimated at 0.7%–1.4% of general population (Makharia et al., 2022). In Europe, a higher prevalence has been reported in northern (1.6%) compared to eastern (0.98%), southern (0.69%), and western (0.60%) countries (Roberts et al., 2021). These data are not dissimilar from those reported in the United States, where Fasano et al. described a 0.8% prevalence in 2003 (Fasano et al., 2003). In India, the estimated prevalence is 1.04% (Makharia et al., 2011) with a geographical gradient from North (where a wheat-based diet is frequent) to South (Ramakrishna et al., 2016). Similar prevalence and gradient have been reported in China (Yuan et al., 2017). Data from the remaining geographical regions are based on the serological prevalence of celiac-specific antibodies rather than biopsy-confirmed CD, but all suggest a prevalence between 0.5% and 2%, with two notable exceptions - in Japan, Fukunaga et al. (Fukunaga et al., 2018) reported a <0.1% prevalence of confirmed CD in a population study involving more than 2,000 subjects. The low prevalence in this country can be attributable to a lower frequency of the HLA-DQ2/DQ8 haplotype (Saito et al., 2000). On the contrary, an African population originally living in Western Sahara, the Saharawi, has been reported to have a particularly high prevalence of CD. In a study involving 989 Saharawi children, the prevalence was 5.6% (Catassi et al., 1999). Possible reasons include a relatively high level of consanguinity, higher frequencies of HLA-DQ2 and -DQ8 genotypes in their general population, and consumption of elevated quantities of gluten (Catassi et al., 1999).

### 1.1 Genetic and immunological determinants of celiac disease

Genetic determinants are the major contributing player to CD susceptibility. To date, the major histocompatibility complex (MHC) region is the most well-known hereditary component that acts as a prerequisite for CD development, and the strongest effects are attributed to the HLA-DQA1 and HLA-DQB1 genes. Furthermore, almost all patients with CD possess specific variants of human leukocyte antigen (HLA) DQ2 or DQ8 heterodimers – 90%–95% of patients with CD have positive haplotype DQ2 (DQA1*0501/DQB1*0201), while 5%–10% have positive haplotype DQ8 (HLA-DQB1*0302) (Ludvigsson and Murray, 2019a). Even though common HLA-DQ2/DQ8 haplotypes increase the risk of the disease sixfold (Volta and Villanacci, 2011), the HLA-DQ2 and HLA-DQ8 haplotypes are not entirely disease-specific, since a significant percentage of people, most of whom do not have celiac disease, carry these alleles. Thus, it follows that haplotypes DQ2 and DQ8 are necessary but not sufficient for the development of CD (Bevan et al., 1999; Wijmenga and Gutierrez-Achury, 2014; Lindfors et al., 2019).

Currently, the CD is characterized as a polygenic disease with a complex, non-MHC pattern of inheritance, involving MHC and non-MHC genes that together affect the genetic risk of developing the disease. It is well-established that 6 MHC and 43 non-MHC loci, including a higher number of independent genetic variants, are associated with disease risk (Dieli-Crimi et al., 2015).

The MHC region, located on 6p21, carries relevant immune function genes associated with most immune-mediated diseases. The MHC region risk factors we mentioned earlier - HLA-DQA1 and HLA-DQB1 - account for about 22% of the heritability of CD (Gutierrez-Achury et al., 2015). The complex peculiarities in this region, characterized mainly by having numerous genes, high polymorphicity, and linkage disequilibrium, made it very difficult to identify new additional risk variants in this region. A few years ago, precise mapping of the MHC region identified new independent risk variants explaining about 2.5%–3% of disease heritability (Gutierrez-Achury et al., 2015). HLA-DPβ1, HLA-B (classic HLA-B*08 and HLA-B*39:06 alleles), and two SNPs, rs1611710, which shows an effect on HLA-F expression, and rs2301226, which shows an effect on B3GALT4 and HLA-DPB1 expression. Thus, it follows that MHC risk variants account for 25% of the heritability of the disease, leaving a significant portion still unexplained.

In the last years, extensive GWAS studies have shed new light on the risk of CD, identifying independent genetic variants at non-HLA loci that could clarify the complex genetics of this disorder (Smyth et al., 2008; Dubois et al., 2010; Trynka et al., 2011; Coleman et al., 2016). In the case of CD, the first GWAS study resulted in the conclusive identification of the first non-HLA-related CD risk locus, the IL2/IL21 region (van Heel et al., 2007). In the following years, subsequent GWAS studies have shown as many as 14 new regions associated with the development of CD (Hunt et al., 2008; Garner et al., 2009). In 2009, Trynka et al. identified additional susceptibility regions in the *REL, OLIG3,* and *TNFAIP3* (Trynka et al., 2009) increasing the number of non-HLA-related loci identified a year later (Dubois et al., 2010). The same research group, using the Immunochip platform in a study of a large cohort from seven geographic regions, revealed 13 new loci associated with the disease (Trynka et al., 2011). In subsequent years, new GWAS analyses have contributed to adding more risk loci to the "non-HLA risk locus family”. There were corresponding studies by Scandinavian groups describing a risk locus involving the *DUSP10* gene after stratification for HLA-DQ risk factors (Östensson et al., 2013) or the Irish group’s research—increasing the total number of common non-HLA CD susceptibility loci by two more (*ZNF335* and *NFIA*) (Coleman et al., 2016).

The group of non-HLA genes has greatly expanded where the majority have been reported to be related to other autoimmune diseases, or those related to T and B cell functions such as antigen presentation and cytokine production (Abadie et al., 2011). These genes are involved in the peptide recognition and CD4^+^ T cell presentation (*HLA-B, HLA-DPB11, HLA-F1HLA-DQA1, HLA-DQB1*) (Gutierrez-Achury et al., 2015) differentiation (*CCR1, CCR2, CCR3, STAT4, PTPN2, RUNX3, THEMIS, ETS1, SH2B3, IL12A, IL18R1, IL18RAP, IL1RL1, IL1RL2, CCR4* (Festen et al., 2011a; Dieli-Crimi et al., 2015), survival (*FASLG, TNFSF18*), migration (*RGS1, ELMO* RGS1, *ITGA4*) (Hunt et al., 2008), activation of T and B cells (*ICOSLG, RGS1, BACH2, POU2AF1, TNFAIP3, ZFP36L1, MAP3K7, IL-21, CCR9*, *RGS1*, *CTLA4, ICOS* ^3^ *, CD28*,^,^ *RGS1, PRKCQ, KIAA1109, ADAD1, IL2, IL21, KIAA1109, ADAD1, IL2, IL21, CTLA4, ICOS, CD28, CD80, PTPN 2, IL2, FASLG, CD247, SH2B3, UBASH3A, PRKCQ, TAGAP, ARHGAP31, RGS13 CTLA4, ICOSLG, RGS1, BACH2, POU2AF1, TNFAIP3* and *ZFP36L1*) (Smyth et al., 2008; Dieli-Crimi et al., 2015) or in antigen presentation (*CD80, TNFSF4, CIITA, ELM01, NFIA*) (Abadie et al., 2011; Meresse et al., 2012). It is currently estimated that the identified MHC genetic variants as well as the remaining discovered non-MHC genetic variants explain about 31% of the heritability of celiac disease. It is noteworthy that non-MHC variants have been estimated to account for 6.5% of CD heritability, which means, a much more important role of the classic, known MHC variants. Thus, it seems that the remaining variants responsible for the largest part of heritability - accounting for practically 70%, are low-effect variants (except for MHC variants) (Dieli-Crimi et al., 2015). Genetic risk variants associated with celiac disease are presented in Supplementary Table S1.

### 1.2 Nutritional determinants of celiac disease

In the development of CD dietary factors are also crucial. These primarily include exposure to gluten; a person who has never consumed gluten will not develop CD (Ludvigsson and Murray, 2019b). However, it is noteworthy that the diagnosis rate of CD has increased in recent years. This is partly explained by access to better diagnostic tools but there is also much evidence of the contribution of environmental and dietary factors (King et al., 2020).

For many years, breastfeeding and the time of introducing gluten into the diet were considered as factors that could affect the risk of developing CD. Even in the recommendations of the British Society of Gastroenterology from 2014, we can read that children who are breastfed during and after the introduction of gluten to the diet may have a lower risk of developing CD and that large amounts of gluten or exposure to gluten in children not breastfed may increase the risk of developing celiac disease (Ludvigsson et al., 2014). However, in the latest 2019 guidelines, the European Society for the Study of Celiac Disease emphasizes that there is no evidence to support the thesis that the time of breastfeeding or the time of introducing gluten into the infant’s diet - at 4 months of age or between 6 and 12 months of age–has an impact on the risk of developing CD (Al-Toma et al., 2019). The results of two studies, PREVENTCD and CELIPREV, are particularly highlighted (Lionetti et al., 2014; Vriezinga et al., 2014).

The first study was a multicenter study conducted by Vriezinga et al. (Vriezinga et al., 2014) on a group of 944 children from 8 countries with HLA-DQ2 or HLA-DQ8 positivity and at least 1 first-degree relative with CD. Children were divided into two random groups–the first group, 475 participants received 100 mg of immunologically active gluten daily between 16 and 24 weeks of age. In the second group, 469 children received a placebo. At 3 years of age, every participant underwent a biopsy to confirm or exclude celiac disease. As compared with a placebo, the introduction of small quantities of gluten at 16–24 weeks of age did not reduce the risk of CD in the group of high-risk children. Also, gluten introduction during breastfeeding did not show any protective effect on CD development. Furthermore, the study revealed that breastfeeding - exclusive as well as any breastfeeding - and duration of breastfeeding did not significantly impact the development of CD (Vriezinga et al., 2014). The second study conducted by Lionetti et al. compared the time of gluten introduction in children born in Italy. Gluten was introduced at 6 months of age in a group of 297 infants or at 12 months of age in a group of 256 infants. All children had a first-degree relative with CD. In this study, the delayed introduction of gluten and breastfeeding did not modify the risk of CD among at-risk infants, although the later introduction of gluten was associated with a delayed onset of disease but without influencing the overall risk (Lionetti et al., 2014).

An interesting issue is also the amount of gluten in a child’s diet in the context of the later development of CD. Three studies (Andrén et al., 2019; Lund-Blix et al., 2019; Mårild et al., 2019) were published in the 2019. Two of them were conducted in the at-risk CD population, and one included children independent of HLA. It was observed that higher gluten consumption in the first years of life was associated with a higher risk of being diagnosed with CD or CD autoimmunity. Ludvigsson comments that taking into account the outcomes of these studies, 2 g of gluten per day which responds to one extra slice of bread seems to be linked to a 20%–50% increased risk of CD (Ludvigsson and Lebwohl, 2020).

In conclusion, there is no evidence that breastfeeding, as well as breastfeeding while introducing gluten into the diet, reduces the risk of developing CD. Also, the timing of introducing gluten into a child’s diet does not seem to affect the development of the disease–ESPGHAN recommends introducing gluten between 4 and 12 months of age although there is no recommendation regarding the type and the amount of gluten to be used at introduction (Szajewska et al., 2015; ESPGHAN, 2016; Silano et al., 2016). At the same time, ESPGHAN suggests avoiding large amounts of gluten during the first month after gluten introduction (ESPGHAN, 2016).

It is also worth noting that our diet and lifestyle have changed significantly over the last few decades. Several links could be made between a Western-style diet (WD) and CD development but this area has yet not been fully investigated. Nevertheless, WD can be characterized as a high-caloric diet, rich in refined grains and sugar, salt, saturated fats, and animal protein, and low in fiber, vitamins, and trace elements (García-Montero et al., 2021). Such a composition of diet could increase the risk of CD contributing to gut dysbiosis and changes in intestinal barrier function. This can increase intestinal permeability, further leading to mucosal inflammation, leakage of toxic bacterial metabolites into the circulation, and finally systemic endotoxemia and chronic inflammation (García-Montero et al., 2021). Since WD is based on processed foods, and low in fresh fruits and vegetables, its anti-inflammatory and antioxidant status is low which also can predispose to low-grade chronic inflammation (Malesza et al., 2021).

Furthermore, Malesza et al. (Malesza et al., 2021) state that changes in microbiota induced by a high-fat diet that is common for Western dietary patterns can also disrupt the expression of inflammation- and metabolism-related genes, reduce short-chain fatty acids (SCFA) production, increase lipopolysaccharide (LPS) production and the activity endocannabinoid system. Authors summarize that a high-fat diet enhances oxidative stress by increasing reactive oxygen species (ROS) and reactive nitrogen species (RNS) production, stimulating closely related ER stress, downregulating gut peptide signaling pathways, and reducing their secretion by enteroendocrine cells (Malesza et al., 2021). A summary of dietary factors associated with CD is presented in Table 1.

**TABLE 1:** Diet-related factors associated with celiac disease.

Factor	Impact	References
Exposure to gluten	Exposure to gluten may activate cell-mediated and humoral immune responses leading to crypt hyperplasia, lymphocyte infiltration, and villous atrophy in the small intestine in genetically predisposed individuals	Ludvigsson and Murray (2019b)
Time of gluten introduction to the diet	The timing of introducing gluten into a child’s diet does not seem to affect the development of the disease	Lionetti et al. (2014), Vriezinga et al. (2014)
Breastfeeding	Exclusive/any breastfeeding, and breastfeeding at the time of gluten introduction, does not reduce the risk of developing CD during childhood	Lionetti et al. (2014), Vriezinga et al. (2014)
Amount of gluten	Higher gluten consumption in the first years of life is associated with a higher risk of CD development	Andrén et al. (2019), Lund-Blix et al. (2019), Mårild et al. (2019)
Western and high-fat diet	Western-style diet possibly could predispose to CD development	García-Montero et al. (2021), Malesza et al. (2021)
	1. Western and high-fat diets impact gut microbiota, driving gut dysbiosis	
	2. Gut dysbiosis results in a reduction of SCFA production, further increasing LPS production and the activity endocannabinoid system, and decreasing antimicrobial Paneth cell peptides	
	3. WD and high-fat diets increase ROS and RNS production, stimulating closely related ER stress, further downregulating gut peptide signaling pathways, and reducing their secretion by enteroendocrine cells	
	4. Reduction in tight junction expression, increased intestinal permeability, leakage of toxic bacterial metabolites into the circulation, systemic endotoxemia, and chronic inflammation	
	5. Dysbiosis and high-fat diet drive activation of TLR4 by LPS and SFA, NF-κB stimulation and production of IL-6 and TNF-alfa, and activation of neutrophils and macrophages. Increased secretion of bile acids can impair gut barrier function and have pro-inflammatory effects	

### 1.3 Gut microbiota and celiac disease

In normal conditions, the gut microbiota includes at least six bacterial phyla: *Firmicutes, Bacteroidetes, Actinobacteria, Proteobacteria, Fusobacteria,* and *Verrucomicrobia* (Arumugam et al., 2011). Changes in the composition and function of gut microbiota have been linked to many gastrointestinal diseases, including CD. Both cross-sectional and cohort-prospective studies investigated the role of the intestinal microbiome in CD.

Cross-sectional studies provided highly heterogeneous results. Limitations of these studies included highly individual-specific microbial profiles, small sample sizes, and spurious “healthy controls” (actually including patients who underwent upper digestive endoscopy for symptoms) (Valitutti et al., 2019). Despite these limitations, a decrease in *Bifidobacteria* and an increase in *Bacteroides* (both on feces and mucosal biopsies) were commonly reported (Valitutti et al., 2019). More reliable information about the dynamic changes in the gut microbiome of CD patients came from prospective studies. In the PROFICEL study, De Palma et al. (Palma et al., 2012) reported modification of the gut microbiota before the actual development of CD. In detail, infants with genetic susceptibility to CD had feces characterized by a higher number of *Bacteroides fragilis* and *Staphylococcus spp*. and a lower number of *Bifidobacteria* and *B. Longum* vs. healthy controls (Palma et al., 2012). The same study group published two additional studies. In the most extensive longitudinal analysis of gut microbiota, Sellitto et al. (Sellitto et al., 2012) examined stool samples at several time points (7 days, 30 days, 6 months, 8 months, 10 months, 12 months, 18 months, and 24 months) in infants. The results of this study suggested relevant differences between the evolving microbiota of infants with a genetic predisposition for CD compared to those from infants with a non-selected genetic background. In detail, children with a genetic predisposition to CD had increased *Firmicutes* and *Proteobacteria*, while *Actinobacteria* and *Bacteroidetes* were significantly restricted. Additionally, they also found that stool microbiota in these infants did not stabilize, nor was it similar to adult microbiota at 1 year of age (Sellitto et al., 2012). In another study examining stool samples from infants at genetic risk within the first week of life, and at 4 months and 6 months of age, a higher number of enterotoxigenic *E. coli* (ETEC) was identified in infants with a high genetic risk versus those of intermediate risk on formula feeding (Olivares et al., 2018). The Celiac Disease Genomic, Environmental, Microbiome, and Metabolomic (CD-GEMM) was another multicenter prospective study investigating blood and stool biomarkers in infants at risk for CD (Leonard et al., 2015). The first paper was published in 2021, reporting longitudinal analyses of gut microbiota, functional pathways, and metabolites, starting from 18 months before CD onset in 10 infants who developed CD and 10 matched nonaffected infants (Leonard et al., 2021). The authors found that the evolving microbiome of CD infants was characterized by an abundance of microbial species and strains that had previously been linked to autoimmune and inflammatory conditions (e.g., *Dialister invisus, Parabacteroides* sp.*, Lachnospiraceae*). On the other hand, a relative lack of other species known to have anti-inflammatory effects (e.g., *Streptococcus thermophilus, Faecalibacterium prausnitzii,* and *Clostridium clostridioforme*) occurred before the diagnosis of CD (Leonard et al., 2021). Gut microbiota changes associated with CD are presented in Table 2.

**TABLE 2:** Gut microbiota changes associated with celiac disease.

Observation 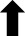	Reference
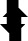 *Bifidobacteria* and an *Bacteroides*	Valitutti et al. (2019)
number of *Bacterioides fragilis* and *Staphylococcus spp*.	Palma et al. (2012)
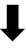 number of *Bifidobacteria* and *B. Longum*	
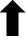 *Firmicutes* and *Proteobacteria*,	Sellitto et al. (2012)
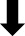 *Actinobacteria* and *Bacteroidetes* were significantly restricted in children with a genetic predisposition to CD	
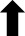 number of enterotoxigenic *E. coli* (ETEC) in infants with a high genetic risk versus those of intermediate risk on formula feeding	Olivares et al. (2018)
microbial species and strains linked to autoimmune and inflammatory conditions (e.g., *Dialister invisus, Parabacteroides sp., Lachnospiraceae*) and relative lack of species with anti-inflammatory effects (e.g., *Streptococcus thermophilus, Faecalibacterium prausnitzii,* and *Clostridium clostridioforme*) before the diagnosis of CD in infants	Leonard et al. (2021)

### 1.4 Environmental determinants of celiac disease

Environmental factors appear to significantly influence the development of CD. Common gastroenterological infections have been shown to increase the risk of developing CD (Kagnoff et al., 1984; Beyerlein et al., 2017; Kemppainen et al., 2017; Mårild et al., 2019).

Studies indicate that enteral viruses in particular are associated with the development of CD. Lindofrs et al. conducted a prospective metagenomics screening of the stool virome in 83 CD genetically predisposed children and 83 controls. They observed that frequent exposure to enterovirus between 1 and 2 years of age was associated with an increased risk of CD autoimmunity. Moreover, they revealed that enteroviruses and higher amounts of gluten in the diet have a cumulative effect on CD development (Lindfors et al., 2020). Similarly, Khar et al. found that a higher frequency of enterovirus, but not adenovirus infections, during early childhood was associated with later CD in a cohort of 220 Norwegian children (Kahrs et al., 2019). Oikarinen et al. confirmed the association observed in two previous studies between enterovirus infections and the later development of CD (Oikarinen et al., 2021). It is also indicated that early-life parechovirus and rotavirus infections are associated with subsequent CD in genetically at-risk children and that also reovirus infection may trigger CD (Stene et al., 2006; Bouziat et al., 2017; Tapia et al., 2021).

It seems that viral infections are involved in immune activation and the breakdown of tolerance against gluten in genetically predisposed individuals. Moreover, viral infections in infants could affect the maturation and development of the mucosal immune system and cause long-term changes in the gut microbiota (Kiliccalan, 2021). An interesting study was conducted by Kemppanien et al. to investigate the relationship between reported infections, rotavirus vaccination status, time to the first introduction of gluten, breastfeeding, and risk of celiac disease autoimmunity in the group of 6327 genetically predisposed children aged 1–4 years from The Environmental Determinants for Diabetes in the Young (TEDDY) study. They observed that gastrointestinal infections increase the risk of CD autoimmunity within the following 3 months by 33% and that the risk is modified by HLA genotype, infant gluten consumption, breastfeeding, and rotavirus vaccination. The risk of developing CD autoimmunity was additionally higher in winter-born infants to whom gluten was introduced before the age of 6 months, and 10 times higher in children without the HLA-DQ2 allele (carrying the HLA-DQ8/8 or HLA-DQ4/8 genotypes) and breastfed for less than 4 months. In contrast, the risk was reduced in children vaccinated against rotavirus who had introduced gluten into their diet before the age of 6 months (Kemppainen et al., 2017). This study shows the cumulative effect of risk factors (Barone and Auricchio, 2021).

Interesting results are also given by studies on bacterial infections pointing to an inverse association between *H. pylori* infection and CD development (Amlashi et al., 2021), although Dore et al. did not find any relationship between *H. pylori* and CD risk (Dore et al., 2018). In turn, Riddle et al. observed an increased risk of CD following Campylobacteriosis (Riddle et al., 2013).

Among environmental factors, the relationship between smoking and the development of CD, as well as the type of delivery, was also examined. It was observed that smokers have a significantly decreased risk of CD compared with non-smokers (Wijarnpreecha et al., 2018). In turn, the mode of delivery was not an independent risk factor for the development of CD autoimmunity or CD in children in TEDDY cohort (Koletzko et al., 2018).

Gaylord et al. (Gaylord et al., 2020) conducted a study to identify whether persistent organic pollutants (POPs) which are endocrine disruptors could be potential risk factors for CD. Authors found higher odds of CD associated with specific POPs, in particular with p,p'-DDE (p,p’-dichlorodiphenyldichloroethylene). This study is the first to highlight the potential role of endocrine disruptors in the development of CD. However, further research is needed in this area.

Environment-related factors associated with celiac disease are presented in Table 3.

**TABLE 3:** Environment-related factors associated with celiac disease.

Factor	Impact	References
Viral infections	The cumulative effect of gliadin and viruses	Stene et al. (2006), Bouziat et al. (2017), Lindfors et al. (2020), Barone and Auricchio (2021), Oikarinen et al. (2021), Tapia et al. (2021)
	Viral infections are involved in immune activation and the breakdown of tolerance against gluten in genetically predisposed individuals	
	In infants, viruses could affect the maturation and development of the mucosal immune system and cause long-term changes in the gut microbiota	
	Viral ligands delay vesicular trafficking, and activate innate immunity, and inflammatory markers, e.g., NFkB and MAPK, activate TLRs	
Bacterial infections	Mixed results	Riddle et al. (2013), Dore et al. (2018), Amlashi et al. (2021)
	Possible mechanism	
	Molecule mimicking; T cell receptor cross-reactivity between gliadin and bacterial peptides	
Smoking	Significantly decreased risk of celiac disease compared with non-smokers	Wijarnpreecha et al. (2018)
Persistent organic pollutant	Higher odds of CD associated with specific persistent organic pollutant	Gaylord et al. (2020)
Type of delivery	Type of delivery is not an independent factor of celiac disease	Koletzko et al. (2018)

## 2 Celiac disease novel therapies

Currently, the only effective form of treatment for CD is a strict gluten-free diet. So far no drugs for celiac disease treatment have been approved by the Food and Drug Administration. However, given the numerous limitations of a gluten-free diet, including cost, reduced quality of life, or lack of response to treatment with a gluten-free diet in up to 7%–30% of patients, new treatment strategies are being sought (Varma and Krishnareddy, 2022).

Refractory CD (RCD) is diagnosed when relapsing symptoms persist despite a strict gluten-free diet (GFD) for more than 12 months and in the absence of other diseases, including overt lymphoma. Treatment of RCD involves a combination of nutritional support and immunosuppressive therapy - steroid therapy, thiopurines infliximab, and mesalamine. However, this treatment is often not effective (Al-Toma et al., 2019). Some patients diagnosed with RCD may respond to trace amounts of gluten in the diet, even below - considered safe for the vast majority of CD patients - 20 ppm. Hollon et al. conducted an interesting study on a group of patients who were non-responsive to GFD treatment. The study involved 17 patients who remained symptomatic despite adhering to a strict gluten-free diet, six of whom were diagnosed with RCD before entering the study. They were then placed on a 3–6 months special diet consisting of unprocessed, whole gluten-free products known as the Gluten Contamination Elimination Diet (GCED). Out of the 17 patients, 14 (82%) responded positively to the GCED. After undergoing GCED, all five previously diagnosed RCD patients became asymptomatic and no longer met the criteria for RCD. Out of the 14 patients who responded to the GCED, 11 (79%) were able to successfully return to a traditional GFD without experiencing a recurrence of symptoms (Hollon et al., 2013).

However, new approaches are being sought to treat CD more effectively and move beyond a strict GFD. One proposed strategy aims to reduce immunogenic gluten peptides through intraluminal digestion. This involves the oral administration of exogenous endopeptidases that digest gluten in the intestinal lumen. This prevents gluten from reaching the lamina propria and stimulating the immune system (Varma and Krishnareddy, 2022). Other proposed strategies aim at blocking immune response to gluten peptides by:

• transglutaminase transglutaminase 2 (TG2) blockers preventing deamidation of gluten peptides and their efficient presentation to CD4^+^ T cells (Paolella et al., 2022);
• inhibiting epithelial damage driven by IL-15 with anti-IL15 antibodies or opposing the outgrowth of malignant IELs in type II refractory CD;
• immunotherapy to restore gluten tolerance through stimulation-induced death of small intestinal epithelial cells and immune activation through the production of regulatory T cells (Cerf-Bensussan and Schuppan, 2021; Varma and Krishnareddy, 2022).

A promising new therapeutic approach is the first TG2 inhibitor in clinical trials, ZED1227, which is an oral selective inhibitor of TG2 (Büchold et al., 2022). In phase 1 clinical studies consumption of 500 mg ZED1227 for up to 8 days turned out to be safe. In phase 2, authors checked in remised patients with CD who were challenged with daily gluten intake - 3 mg of gluten - for 6 weeks, if exposure to ZED1227 prevents symptoms from recurring. The trial was a randomized, double-blind, placebo-controlled, dose-finding study. Authors found that the ZED1227 effectively attenuated gluten-induced intestinal mucosal injury (Schuppan et al., 2021).

TAK-101, a gliadin encapsulation in negatively charged poly (DL-lactide-glycolic acid) nanoparticles, is another promising approach. In a phase 2 study, 33 patients with CD underwent a 14-day gluten challenge to assess whether TAK-101 induces gluten-specific tolerance. The study found that the drug resulted in an 88% reduction in interferon-γ spot-forming units compared to the placebo (2.01 vs. 17.58, *p* = .006). Additionally, TAK-101 reduced changes in circulating α4β7+CD4^+^ (0.26 vs. 1.05, *p* = .032), αEβ7+CD8^+^ (0.69 vs. 3.64, *p* = .003), and γδ (0.15 vs. 1.59, *p* = .010) effector memory T cells. TAK-101 was well tolerated and prevented gluten-induced immune activation, so this immunotherapy shows potential for CD treatment and requires further clinical development (Kelly et al., 2021).

In addition, researchers focus on investigating modulators of tight junctions, known as zonula occludens, regulating intestinal permeability which is increased in CD patients resulting in the activation of immune response to indigestible gluten peptides. This process is mediated by a key tight junction modulator–zonulin. Production of zonulin is induced–mainly–by bacteria overgrowth and gluten that binds to receptor CXCR3 in erythrocytes (Fasano, 2020; Machado, 2023). On the other hand, zonuline activates tight junction relaxation, causing the delivery of gliadin peptides to lamina propria. The therapeutic approach targeting zonulin seems to be promising since intestinal permeability is theorized to be an initial promoting event in the etiologic of CD (Hoilat et al., 2022).

One of the zonulin inhibitors, that blocks its receptor and acts as an anti-zonulin receptor inhibitor, is larazotide acetate also known as AT-1001 - a novel, eight-amino acids peptide (Hoilat et al., 2022). Larazotide acetate rebuilds the disturbed tight junction complex, preventing the intestinal permeation of gliadin (Slifer et al., 2021).

Larazotide acetate in phase I and II studies was shown to be safe, well tolerated and to prevent worsening of gluten-induced symptom severity and to suppress serological markers. However, a placebo-controlled phase III study was terminated (Varma and Krishnareddy, 2022).

A meta-analysis of four trials, including a total of 626 patients, indicates that larazotide acetate is safe and more effective than placebo in alleviating gastrointestinal symptoms in patients with celiac disease who are challenged with gluten. However, it is considered more of a supplement to a gluten-free diet rather than a replacement for it (Hoilat et al., 2022).

Moreover, an important role in the degradation of intestinal villi in CD patients appears to be IL-15, which is an inflammation-stimulating cytokine. A study using the first anti-IL-15 monoclonal antibodies - AMG 714 - was conducted by Lähdeaho et al. on a group of 64 patients with CD (Lähdeaho et al., 2019). In a randomized, double-blind, placebo-controlled, parallel-group study, 150 mg and 300 mg of AMG 714 compared with placebo in adults with CD after controlled gluten provocation, there was no statistically significant difference in change in villous height to crypt depth ratio from baseline after 12 weeks of treatment. However, at the 300 mg dose, authors observed alleviation of some symptoms in response to gluten ingestion assessed by lower - than at the 150 mg and placebo dose - intraepithelial lymphocyte density, patient-reported outcomes, and diarrhea. The authors indicate that the study suggests that the inhibition of IL-15 is a viable strategy in the treatment of CD and point to the need for further studies on non-responsive to gluten-free diet CD (Lähdeaho et al., 2019).

Trials have been also conducted on antigen-specific immunotherapy. Nexvax2 is a therapeutic vaccine that contains three gluten peptides derived from wheat, barley, and rye, including HLA-DQ2-restricted epitopes commonly recognized by gluten-specific T-cells. However, studies have shown that the vaccine did not achieve the desired effect of reducing symptoms caused by gluten consumption and did not increase tolerance to gluten peptides (Goel et al., 2017).

**FIGURE 1:**
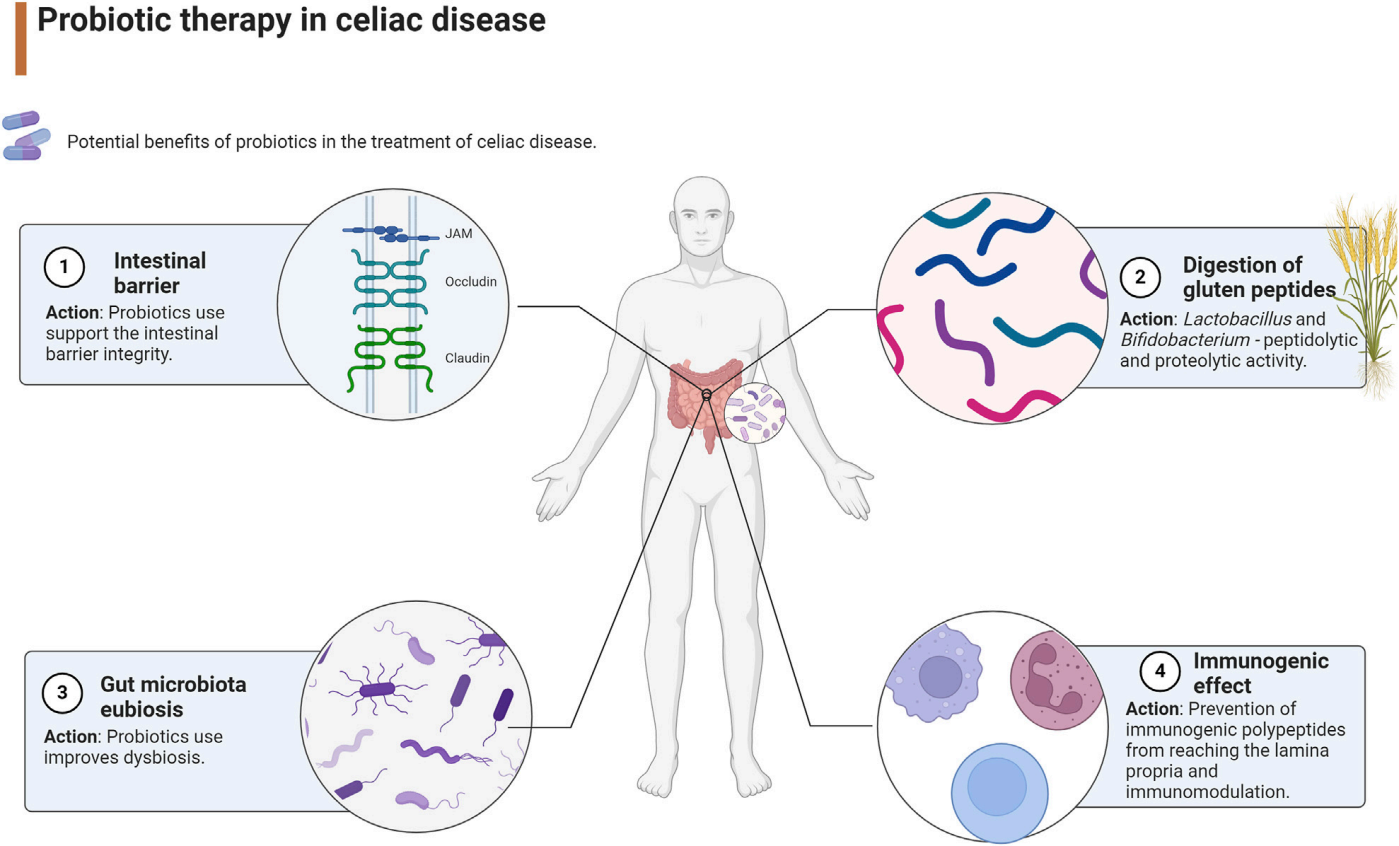
Probiotic therapy in celiac disease (Kõiv and Tenson, 2021).

Moreover, novel therapies include probiotic therapy that potentially may improve gut microbiota composition and maintain gut microbiota homeostasis, digest gluten peptides into small polypeptides, and limit the availability of immunogenic polypeptides to lamina propria (Krishnareddy, 2019; Varma and Krishnareddy, 2022). The potential benefits of probiotics in the treatment of celiac disease are presented in Figure 1.

Reviews of studies indicate that probiotics may improve gastrointestinal symptoms in patients with CD, moderate the immune response, and improve dysbiosis in patients with CD and autoimmune CD. However, high-quality clinical trials are needed to increase the certainty of the evidence (Seiler et al., 2020; Mozafarybazargany et al., 2023). The positive impact of probiotics on CD is primarily attributed to their ability to improve the tightness of the intestinal barrier. Moreover, studies have shown that bacteria from the *Lactiplantibacillus* and *Bifidobacterium* genera, which possess extensive peptidolytic and proteolytic activity, are particularly effective in breaking down gluten compared to other intestinal bacteria (Moawad et al., 2023).

In 2023 Khorzoghi et al. observed that 12-week treatment with a probiotic combination containing *Bifidobacterium and Lactiplantobacillus* species *and S. thermophilus* resulted in a reduction in the intensity of CD clinical symptoms - fatigue, muscle discomfort, bloating, and a gassy feeling - compared to placebo (Soheilian Khorzoghi et al., 2023).

However, it seems that probiotics are not seen as a promise for a quick cure, but rather as a supplement to alleviate the severity and symptoms (Kõiv and Tenson, 2021).

Moreover, endopeptidases of several *Lactobacillus* species–*L. ruminus, L. john donne, L. amylovorus, L. salivarius, L. alimentaris, L. brevis, L. sanfranciscenis and L. hilgardi*–can degrade gluten peptides when added to the starter culture for wheat bread production. This presents promising opportunities for the practical application of these strains in gluten-free food production.

In Table 4 we present ongoing and completed clinical trials concerning pharmaceutical treatment of CD.

**TABLE 4:** Completed and ongoing clinical trials concerning celiac disease novel treatment. Based on Alhassan E et al. Cell Mol Gastroenterol Hepatol. 2019; 8 (3):335–345. doi:10.1016/j.jcmgh. 2019.04.017 and Varma et al. Drugs. 2022 October; 82 (15):1515–1526. doi: 10.1007/s40265-022-01784–2. Epub 2022 October 17. PMID: 36251239.

Status	Drug	Therapeutic approach	Clinical trial number (trial phase)	Outcomes summary
Completed	1) IMGX003 (Latiglutenase)	1) Degradation of gluten peptides; peptidase therapy	1) NCT00859391 (0)	1) Reduction of gluten-induced intestinal mucosal damage and symptom severity (NCT03585478)
	2) AN-PEP	Reduction of immunogenic potential of gluten	NCT00959114 (2a)	2) No effect in preventing mucosal damage after consumption of 7 g of gluten per day for 2 weeks
	3) BL-7010	2) Endopeptidase derived from the fungus Aspergillus niger	NCT00669825 (I)	3) Not available
	4) STAN-1	3) Prevention of gliadin breakdown into immunogenic peptides	NCT01255696 (IIa)	4) No significant difference in tTG-IgA concentration between groups: STAN-1 vs. placebo for 12 weeks + gluten 1 g/day
	5) KAN-101	4) Gluten degradation before absorption	NCT01917630 (Iib)	5) Not available
	6) Necator americanus inoculation	5) Antigen-specific immune tolerance, tolerogenic immunotherapy	NCT03585478 (II)	6) Symptom improvement, no changes in intraepithelial lymphocyte counts and Marsh scores, reduction in intestinal T cells expressing IFN-γ after hookworm infection with an increase in CD4 (+) Foxp3 (+) regulatory T cells
	7) Nexvax2	6) Gluten tolerization	2) NCT00810654 (I/II)	7) Well tolerated, discontinued — not significant improvement
	8) TIMP-GLIA (CNP-101)	7) Gluten vaccine and tolerization	NCT02060864 (I)	8) Well-tolerated, prevention of gluten-induced activation
	9) AMG 714	8) Immune gluten tolerization	NCT01335503 (I)	9) Not available
	10) Larazotide acetate (or AT-1001	9) Anti-IL-15 monoclonal antibody	3) NCT01990885 (I/II)	10) Symptom improvement, lack on data on histologic improvement
	11) TAK062	10) Tigh junction modulator, prevention of gliadin-induced permeability, reduction of small intestinal inflammation	4) NCT00962182 (I/II)	11) Safe and well-tolerated
	12) RO5459072	11) Gluten degradation	5) NCT04248855 (I)	12) Not available
	13) Hu-Mik-Beta-1	12) Inhibition of cathepsin S	6) NCT00671138 (I/II)	13) Not available
		13) Cytokine receptor antibodies	NCT00671138 (II)	
			NCT02754609 (I)	
			7) NCT02528799 (I)	
			NCT03644069 (II)	
			NCT03543540 (I)	
			NCT00879749 (I)	
			8) NCT03738475 (Iia), NCT03486990	
			9) NCT02637141/NCT02633020/NCT03439475	
			10) NCT01396213 (Iib)	
			NCT00386165 (I)	
			NCT00492960 (II)	
			NCT00362856 (II)	
			NCT00386490 (I)	
			NCT00889473 (II)	
			NCT00620451 (II)	
			11) NCT03701555 (I)	
			12) NCT02679014	
			13) NCT01893775 (I)	
Ongoing	1) TAK062	1) Gluten degradation	1)NCT05353985(II)	
	2) KAN-101	2) Tolerogenic immunotherapy	2) NCT05574010 (1/2)	
	3) AN-PEP	3) Endopeptidase	2) NCT05574010	
	4) Latiglutenase	4) Peptidase therapy	3) NCT04788797	
	5) TAK-101	5) Gluten degradation	(IV)	
	6) Teriflunomide	6) Adaptive T cell activation	4) NCT04839575 (II)/NCT04243551 (II)/	
	7) Deamidation and sequestration	7) Gluten sequestration	5) NCT04530123 (II)	
	AGY	8) Anti-IL-23 monoclonal	6) NCT04806737	
	8) Immune targets	Antibody	7) NCT03707730 (II)	
	Guselkumab	9) Anti-IL-15 monoclonal	8) NCT04704843 (Ib)	
	9) PRV-015 Anti-IL-15 monoclonal antibody	Antibody	9) NCT04424927	
	10) PTG-100	10) A4b7 integrin antagonist	(IIb)	
			10) NCT04524221 (Ib)	

Another proposed approach, currently at the experimental stage, is to bind gluten and prevent its further metabolism using poly (hydroxyethylmethacrylate-co-styrenesulfonate). This method has been shown to reduce the digestion of wheat gluten and barley hordein, as well as attenuate the immune response to gluten in food mixtures in rodents (Pinier et al., 2012). In contrast, Kaperchan et al. proposed a series of gluten peptides in which the proline residues were replaced by azidoprolines. These peptides bind to HLA-DQ2 with an affinity similar to that of the natural gluten peptide. Some of these peptides are non-immunogenic and block gluten-induced immune responses. Therefore, they could potentially be used to develop HLA-DQ2-blocking peptides (Kapoerchan et al., 2008).

Technolodzy próbują też wykorzystać możliwości modyfikacji genetycznej to reduce immunotoxic components of gluten (Ghazanfar et al., 2023; PubMed, 2024).

## 3 Conclusion

Celiac disease is an immune-mediated disorder influenced by genetic variants, with MHC variants explaining most of the heritability of CD. In addition to genetic factors, external factors also play a role in increasing the risk of the disease.

Nutritional factors are one such external factor, however, while several links have been suggested between a Western-style diet and CD development, this area has not yet been fully investigated. It is recommended to avoid large amounts of gluten in the first month after gluten introduction, but there is no evidence to support the protective properties of breastfeeding or the timing of gluten introduction. The composition and function of gut microbiota have been linked to CD and common gastroenterological infections have been shown to increase the risk of developing CD. The type of delivery is not an independent factor in the development of CD or CD autoimmunity. What is interesting, it has been found that smokers have a significantly lower risk of developing CD compared to non-smokers.

Currently, the GFD is the only widely accepted treatment for CD, although there is ongoing research for novel therapies. The investigations focus on reducing immunogenic gluten peptides, blocking the immune response to gluten peptides, and immunotherapy to restore gluten tolerance. Novel therapies also include probiotic therapy and modulators of tight junctions that regulate intestinal permeability. It is thought that a cure for CD, which would offer an alternative to the gluten-free diet with its many restrictions, is becoming more attainable as our understanding of the causes and factors of CD increases. However, most of the available options appear to complement a gluten-free diet and offer the opportunity to improve gastrointestinal symptoms among patients, rather than being a direct substitute for a gluten-free diet.

## 4 Statement and declarations

Kinga Skoracka and Anna Maria Rychter are participants of STER Internationalisation of Doctoral Schools Programme from NAWA Polish National Agency for Academic Exchange No. PPI/STE/2020/1/00014/DEC/02.

## References

[B1] Abadie V. Sollid L. M. Barreiro L. B. Jabri B. (2011). Integration of genetic and immunological insights into a model of celiac disease pathogenesis. Annu. Rev. Immunol. 29, 493–525. 10.1146/annurev-immunol-040210-092915 21219178

[B2] Akhabir L. Sandford A. (2010). Genetics of interleukin 1 receptor-like 1 in immune and inflammatory diseases. Curr. Genomics 11 (8), 591–606. 10.2174/138920210793360907 21629437 PMC3078684

[B3] Al-Toma A. Volta U. Auricchio R. Castillejo G. Sanders D. S. Cellier C. (2019). European Society for the Study of Coeliac Disease (ESsCD) guideline for coeliac disease and other gluten-related disorders. United Eur. Gastroenterol. J. 7 (5), 583–613. 10.1177/2050640619844125 PMC6545713 31210940

[B4] Amlashi F. I. Norouzi Z. Sohrabi A. Shirzad-Aski H. Norouzi A. Ashkbari A. (2021). A systematic review and meta-analysis for association of *Helicobacter pylori* colonization and celiac disease. PLoS One 16 (3), e0241156. 10.1371/journal.pone.0241156 33657108 PMC7928511

[B5] Andrén A. C. Lee H. S. Hård af Segerstad E. M. Uusitalo U. Yang J. Koletzko S. (2019). Association of gluten intake during the first 5 Years of life with incidence of celiac disease autoimmunity and celiac disease among children at increased risk. JAMA 322 (6), 514–523. 10.1001/jama.2019.10329 31408136 PMC6692672

[B6] Arumugam M. Raes J. Pelletier E. Le Paslier D. Yamada T. Mende D. R. (2011). Enterotypes of the human gut microbiome. Nature 473 (7346), 174–180. 10.1038/nature09944 21508958 PMC3728647

[B7] Bakker O. B. Ramírez-Sánchez A. D. Borek Z. A. de Klein N. Li Y. Modderman R. (2021). Potential impact of celiac disease genetic risk factors on T cell receptor signaling in gluten-specific CD4+ T cells. Sci. Rep. 11, 9252. 10.1038/s41598-021-86612-5 33927210 PMC8085175

[B8] Balasopoulou A. Stanković B. Panagiotara A. Nikčevic G. Peters B. A. John A. (2016). Novel genetic risk variants for pediatric celiac disease. Hum. Genomics 10 (1), 34. 10.1186/s40246-016-0091-1 27836013 PMC5105295

[B9] Barone M. V. Auricchio S. (2021). A cumulative effect of food and viruses to trigger celiac disease (CD): a commentary on the recent literature. Int. J. Mol. Sci. 22 (4), 2027. 10.3390/ijms22042027 33670760 PMC7922374

[B10] Bevan S. Popat S. Braegger C. P. Busch A. O'Donoghue D. Falth-Magnusson K. (1999). Contribution of the MHC region to the familial risk of coeliac disease. J. Med. Genet. 36 (9), 687–690. 10507725 PMC1734425

[B11] Beyerlein A. Donnachie E. Ziegler A. G. (2017). Infections in early life and development of celiac disease. Am. J. Epidemiol. 186 (11), 1277–1280. 10.1093/aje/kwx190 28637333

[B12] Bondar C. Plaza-Izurieta L. Fernandez-Jimenez N. Irastorza I. Withoff S. Wijmenga C. (2014). THEMIS and PTPRK in celiac intestinal mucosa: coexpression in disease and after *in vitro* gliadin challenge. Eur. J. Hum. Genet. 22 (3), 358–362. 10.1038/ejhg.2013.136 23820479 PMC3925264

[B13] Bouziat R. Hinterleitner R. Brown J. J. Stencel-Baerenwald J. E. Ikizler M. Mayassi T. (2017). Reovirus infection triggers inflammatory responses to dietary antigens and development of celiac disease. Science. 356 (6333), 44–50. 10.1126/science.aah5298 28386004 PMC5506690

[B14] Büchold C. Hils M. Gerlach U. Weber J. Pelzer C. Heil A. (2022). Features of ZED1227: the first-in-class tissue transglutaminase inhibitor undergoing clinical evaluation for the treatment of celiac disease. Cells 11 (10), 1667. 10.3390/cells11101667 35626704 PMC9139979

[B15] Catassi C. Ratsch I. M. Gandolfi L. Pratesi R. Fabiani E. El Asmar R. (1999). Why is coeliac disease endemic in the people of the Sahara? Lancet 354 (9179), 647–648. 10.1016/S0140-6736(99)02609-4 10466670

[B16] Cerf-Bensussan N. Schuppan D. (2021). The promise of novel therapies to abolish gluten immunogenicity in celiac disease. Gastroenterology 161 (1), 21–24. 10.1053/j.gastro.2021.04.031 33891951

[B17] Coleman C. Quinn E. M. Ryan A. W. Conroy J. Trimble V. Mahmud N. (2016). Common polygenic variation in coeliac disease and confirmation of ZNF335 and NIFA as disease susceptibility loci. Eur. J. Hum. Genet. 24 (2), 291–297. 10.1038/ejhg.2015.87 25920553 PMC4717209

[B18] Dieli-Crimi R. Cénit M. C. Núñez C. (2015). The genetics of celiac disease: a comprehensive review of clinical implications. J. Autoimmun. 64, 26–41. 10.1016/j.jaut.2015.07.003 26194613

[B19] Discepolo V. Lania G. Ten Eikelder M. L. G. Nanayakkara M. Sepe L. Tufano R. (2021). Pediatric celiac disease patients show alterations of dendritic cell shape and actin rearrangement. Int. J. Mol. Sci. 22 (5), 2708. 10.3390/ijms22052708 33800150 PMC7962447

[B20] Dore M. P. Salis R. Loria M. F. Villanacci V. Bassotti G. Pes G. M. (2018). *Helicobacter pylori* infection and occurrence of celiac disease in subjects HLA-DQ2/DQ8 positive: a prospective study. Helicobacter 23 (2), e12465. 10.1111/hel.12465 29345406

[B21] Dubois P. C. A. Trynka G. Franke L. Hunt K. A. Romanos J. Curtotti A. (2010). Multiple common variants for celiac disease influencing immune gene expression. Nat. Genet. 42 (4), 295–302. 10.1038/ng.543 20190752 PMC2847618

[B22] ESPGHAN (2016). Gluten introduction and the risk of coeliac disease | ESPGHAN. Available at: http://www.espghan.org/knowledge-center/publications/Nutrition/2016_Gluten_Introduction_and_the_Risk_of_Coeliac_Disease (Accessed August 21, 2022).

[B23] Fasano A. (2020). All disease begins in the (leaky) gut: role of zonulin-mediated gut permeability in the pathogenesis of some chronic inflammatory diseases. F1000Res 9, . 10.12688/f1000research.20510.1 PMC6996528 32051759

[B24] Fasano A. Berti I. Gerarduzzi T. Not T. Colletti R. B. Drago S. (2003). Prevalence of celiac disease in at-risk and not-at-risk groups in the United States: a large multicenter study. Arch. Intern Med. 163 (3), 286–292. 10.1001/archinte.163.3.286 12578508

[B25] Festen E. A. M. Goyette P. Green T. Boucher G. Beauchamp C. Trynka G. (2011a). A meta-analysis of genome-wide association scans identifies IL18RAP, PTPN2, TAGAP, and PUS10 as shared risk loci for crohn’s disease and celiac disease. PLOS Genet. 7 (1), e1001283. 10.1371/journal.pgen.1001283 21298027 PMC3029251

[B26] Festen E. A. M. Goyette P. Green T. Boucher G. Beauchamp C. Trynka G. (2011b). A meta-analysis of genome-wide association scans identifies IL18RAP, PTPN2, TAGAP, and PUS10 as shared risk loci for Crohn’s disease and celiac disease. PLoS Genet. 7 (1), e1001283. 10.1371/journal.pgen.1001283 21298027 PMC3029251

[B27] Fukunaga M. Ishimura N. Fukuyama C. Izumi D. Ishikawa N. Araki A. (2018). Celiac disease in non-clinical populations of Japan. J. Gastroenterol. 53 (2), 208–214. 10.1007/s00535-017-1339-9 28389733

[B28] García-Montero C. Fraile-Martínez O. Gómez-Lahoz A. M. Pekarek L. Castellanos A. J. Noguerales-Fraguas F. (2021). Nutritional components in western diet versus mediterranean diet at the gut microbiota–immune system interplay. Implications for health and disease. Nutrients 13 (2), 699. 10.3390/nu13020699 33671569 PMC7927055

[B29] Garner C. Ahn R. Ding Y. C. Steele L. Stoven S. Green P. H. (2014). Genome-Wide association study of celiac disease in north America confirms FRMD4B as new celiac locus. PLoS One 9 (7), e101428. 10.1371/journal.pone.0101428 24999842 PMC4084811

[B30] Garner C. P. Murray J. A. Ding Y. C. Tien Z. van Heel D. A. Neuhausen S. L. (2009). Replication of celiac disease UK genome-wide association study results in a US population. Hum. Mol. Genet. 18 (21), 4219–4225. 10.1093/hmg/ddp364 19648293 PMC2758145

[B31] Gaylord A. Trasande L. Kannan K. Thomas K. M. Lee S. Liu M. (2020). Persistent organic pollutant exposure and celiac disease: a pilot study. Environ. Res. 186, 109439. 10.1016/j.envres.2020.109439 32409013

[B32] Gell G. Kovács K. Veres G. Korponay-Szabó I. R. Juhász A. (2017). Characterization of globulin storage proteins of a low prolamin cereal species in relation to celiac disease. Sci. Rep. 7 (1), 39876. 10.1038/srep39876 28051174 PMC5209737

[B33] Ghazanfar H. Javed N. Lee S. Shaban M. Cordero D. Acherjee T. (2023). Novel therapies for celiac disease: a clinical review article. Cureus 15 (5), e39004. 10.7759/cureus.39004 37323330 PMC10263194

[B34] Goel G. King T. Daveson A. J. Andrews J. M. Krishnarajah J. Krause R. (2017). Epitope-specific immunotherapy targeting CD4-positive T cells in coeliac disease: two randomised, double-blind, placebo-controlled phase 1 studies. Lancet Gastroenterol. Hepatol. 2 (7), 479–493. 10.1016/S2468-1253(17)30110-3 28506538 PMC5676538

[B35] G T. Ka H. Na B. Romanos J. Mistry V. Szperl A. (2011). Dense genotyping identifies and localizes multiple common and rare variant association signals in celiac disease. Nat. Genet. 43 (12), 1193–1201. 10.1038/ng.998 22057235 PMC3242065

[B36] Guo C. C. Wang M. Cao F. D. Huang W. H. Xiao D. Ye X. G. (2016). Meta-analysis on associations of RGS1 and IL12A polymorphisms with celiac disease risk. Int. J. Mol. Sci. 17 (4), 457. 10.3390/ijms17040457 27043536 PMC4848913

[B37] Gutierrez-Achury J. Zhernakova A. Pulit S. L. Trynka G. Hunt K. A. Romanos J. (2015). Fine mapping in the MHC region accounts for 18% additional genetic risk for celiac disease. Nat. Genet. 47 (6), 577–578. 10.1038/ng.3268 25894500 PMC4449296

[B38] Hadjadj J. Castro C. N. Tusseau M. Stolzenberg M. C. Mazerolles F. Aladjidi N. (2020). Early-onset autoimmunity associated with SOCS1 haploinsufficiency. Nat. Commun. 11, 5341. 10.1038/s41467-020-18925-4 33087723 PMC7578789

[B39] Hoilat G. J. Altowairqi A. K. Ayas M. F. Alhaddab N. T. Alnujaidi R. A. Alharbi H. A. (2022). Larazotide acetate for treatment of celiac disease: a systematic review and meta-analysis of randomized controlled trials. Clin. Res. Hepatology Gastroenterology 46 (1), 101782. 10.1016/j.clinre.2021.101782 34339872

[B40] Hollon J. R. Cureton P. A. Martin M. L. Puppa E. L. L. Fasano A. (2013). Trace gluten contamination may play a role in mucosal and clinical recovery in a subgroup of diet-adherent non-responsive celiac disease patients. BMC Gastroenterol. 13, 40. 10.1186/1471-230X-13-40 23448408 PMC3598839

[B41] Huang S. Q. Zhang N. Zhou Z. X. Huang C. C. Zeng C. L. Xiao D. (2017). Association of lpp and TAGAP polymorphisms with celiac disease risk: a meta-analysis. Int. J. Environ. Res. Public Health 14 (2), 171. 10.3390/ijerph14020171 28208589 PMC5334725

[B42] Hunt K. A. Mistry V. Bockett N. A. Ahmad T. Ban M. Barker J. N. (2013). Negligible impact of rare autoimmune-locus coding-region variants on missing heritability. Nature 498 (7453), 232–235. 10.1038/nature12170 23698362 PMC3736321

[B43] Hunt K. A. Zhernakova A. Turner G. Heap G. A. R. Franke L. Bruinenberg M. (2008). Newly identified genetic risk variants for celiac disease related to the immune response. Nat. Genet. 40 (4), 395–402. 10.1038/ng.102 18311140 PMC2673512

[B44] Kagnoff M. F. Austin R. K. Hubert J. J. Bernardin J. E. Kasarda D. D. (1984). Possible role for a human adenovirus in the pathogenesis of celiac disease. J. Exp. Med. 160 (5), 1544–1557. 10.1084/jem.160.5.1544 6491604 PMC2187489

[B45] Kahrs C. R. Chuda K. Tapia G. Stene L. C. Mårild K. Rasmussen T. (2019). Enterovirus as trigger of coeliac disease: nested case-control study within prospective birth cohort. BMJ 364, l231. 10.1136/bmj.l231 30760441 PMC6372922

[B46] Kapoerchan V. V. Wiesner M. Overhand M. van der Marel G. A. Koning F. Overkleeft H. S. (2008). Design of azidoproline containing gluten peptides to suppress CD4+ T-cell responses associated with celiac disease. Bioorg Med. Chem. 16 (4), 2053–2062. 10.1016/j.bmc.2007.10.091 18037302

[B47] Kelly C. P. Murray J. A. Leffler D. A. Getts D. R. Bledsoe A. C. Smithson G. (2021). TAK-101 nanoparticles induce gluten-specific tolerance in celiac disease: a randomized, double-blind, placebo-controlled study. Gastroenterology 161 (1), 66–80.e8. 10.1053/j.gastro.2021.03.014 33722583 PMC9053078

[B48] Kemppainen K. M. Lynch K. F. Liu E. Lönnrot M. Simell V. Briese T. (2017). Factors that increase risk of celiac disease autoimmunity after a gastrointestinal infection in early life. Clin. Gastroenterol. Hepatol. 15 (5), 694–702.e5. 10.1016/j.cgh.2016.10.033 27840181 PMC5576726

[B49] Kiliccalan I. (2021). Is the rotavirus vaccine really associated with a decreased risk of developing celiac and other autoimmune diseases? Rambam Maimonides Med. J. 12 (4), e0031. 10.5041/RMMJ.10450 34449304 PMC8549836

[B50] King J. A. Jeong J. Underwood F. E. Quan J. Panaccione N. Windsor J. W. (2020). Incidence of celiac disease is increasing over time: a systematic review and meta-analysis. Am. J. Gastroenterol. 115 (4), 507–525. 10.14309/ajg.0000000000000523 32022718

[B51] Klobuch S. Lim J. J. van Balen P. Kester M. G. D. de Klerk W. de Ru A. H. (2022). Human T cells recognize HLA-DP–bound peptides in two orientations. Proc. Natl. Acad. Sci. 119 (49), e2214331119. 10.1073/pnas.2214331119 36442096 PMC9894132

[B52] Kõiv V. Tenson T. (2021). Gluten-degrading bacteria: availability and applications. Appl. Microbiol. Biotechnol. 105 (8), 3045–3059. 10.1007/s00253-021-11263-5 33837830 PMC8053163

[B53] Koletzko S. Lee H. S. Beyerlein A. Aronsson C. A. Hummel M. Liu E. (2018). Cesarean section on the risk of celiac disease in the offspring: the teddy study. J. Pediatr. Gastroenterol. Nutr. 66 (3), 417–424. 10.1097/MPG.0000000000001682 28753178 PMC5787038

[B54] Krishnareddy S. (2019). The microbiome in celiac disease. Gastroenterol. Clin. North Am. 48 (1), 115–126. 10.1016/j.gtc.2018.09.008 30711204

[B55] Kumar V. Gutierrez-Achury J. Kanduri K. Almeida R. Hrdlickova B. Zhernakova D. V. (2015). Systematic annotation of celiac disease loci refines pathological pathways and suggests a genetic explanation for increased interferon-gamma levels. Hum. Mol. Genet. 24 (2), 397–409. 10.1093/hmg/ddu453 25190711

[B56] Lähdeaho M. L. Scheinin M. Vuotikka P. Taavela J. Popp A. Laukkarinen J. (2019). Safety and efficacy of AMG 714 in adults with coeliac disease exposed to gluten challenge: a phase 2a, randomised, double-blind, placebo-controlled study. Lancet Gastroenterol. Hepatol. 4 (12), 948–959. 10.1016/S2468-1253(19)30264-X 31494096

[B57] Leonard M. M. Camhi S. Huedo-Medina T. B. Fasano A. (2015). Celiac disease genomic, environmental, microbiome, and metabolomic (CDGEMM) study design: approach to the future of personalized prevention of celiac disease. Nutrients 7 (11), 9325–9336. 10.3390/nu7115470 26569299 PMC4663598

[B58] Leonard M. M. Valitutti F. Karathia H. Pujolassos M. Kenyon V. Fanelli B. (2021). Microbiome signatures of progression toward celiac disease onset in at-risk children in a longitudinal prospective cohort study. Proc. Natl. Acad. Sci. U. S. A. 118 (29), e2020322118. 10.1073/pnas.2020322118 34253606 PMC8307711

[B59] Lindfors K. Ciacci C. Kurppa K. Lundin K. E. A. Makharia G. K. Mearin M. L. (2019). Coeliac disease. Nat. Rev. Dis. Prim. 5 (1), 3. 10.1038/s41572-018-0054-z 30631077

[B60] Lindfors K. Lin J. Lee H. S. Hyöty H. Nykter M. Kurppa K. (2020). Metagenomics of the faecal virome indicate a cumulative effect of enterovirus and gluten amount on the risk of coeliac disease autoimmunity in genetically at risk children: the TEDDY study. Gut 69 (8), 1416–1422. 10.1136/gutjnl-2019-319809 31744911 PMC7234892

[B61] Lionetti E. Castellaneta S. Francavilla R. Pulvirenti A. Tonutti E. Amarri S. (2014). Introduction of gluten, HLA status, and the risk of celiac disease in children. N. Engl. J. Med. 371 (14), 1295–1303. 10.1056/NEJMoa1400697 25271602

[B62] Ludvigsson J. F. Bai J. C. Biagi F. Card T. R. Ciacci C. Ciclitira P. J. (2014). Diagnosis and management of adult coeliac disease: guidelines from the British Society of Gastroenterology. Gut 63 (8), 1210–1228. 10.1136/gutjnl-2013-306578 24917550 PMC4112432

[B63] Ludvigsson J. F. Lebwohl B. (2020). Three papers indicate that amount of gluten play a role for celiac disease – but only a minor role. Acta Paediatr. 109 (1), 8–10. 10.1111/apa.15057 31701547

[B64] Ludvigsson J. F. Murray J. A. (2019a). Epidemiology of celiac disease. Gastroenterol. Clin. North Am. 48 (1), 1–18. 10.1016/j.gtc.2018.09.004 30711202

[B65] Ludvigsson J. F. Murray J. A. (2019b). Epidemiology of celiac disease. Gastroenterology Clin. N. Am. 48 (1), 1–18. 10.1016/j.gtc.2018.09.004 30711202

[B66] Lund-Blix N. A. Mårild K. Tapia G. Norris J. M. Stene L. C. Størdal K. (2019). Gluten intake in early childhood and risk of celiac disease in childhood: a nationwide cohort study. Am. J. Gastroenterol. 114 (8), 1299–1306. 10.14309/ajg.0000000000000331 31343439

[B67] Machado M. V. (2023). New developments in celiac disease treatment. Int. J. Mol. Sci. 24 (2), 945. 10.3390/ijms24020945 36674460 PMC9862998

[B68] Makharia G. K. Chauhan A. Singh P. Ahuja V. (2022). Review article: epidemiology of coeliac disease. Aliment. Pharmacol. Ther. 56 (Suppl. 1), S3–S17. 10.1111/apt.16787 35815830

[B69] Makharia G. K. Verma A. K. Amarchand R. Bhatnagar S. Das P. Goswami A. (2011). Prevalence of celiac disease in the northern part of India: a community based study. J. Gastroenterology Hepatology 26 (5), 894–900. 10.1111/j.1440-1746.2010.06606.x 21182543

[B70] Malesza I. J. Malesza M. Walkowiak J. Mussin N. Walkowiak D. Aringazina R. (2021). High-fat, western-style diet, systemic inflammation, and gut microbiota: a narrative review. Cells 10 (11), 3164. 10.3390/cells10113164 34831387 PMC8619527

[B71] Mårild K. Dong F. Lund-Blix N. A. Seifert J. Barón A. E. Waugh K. C. (2019). Gluten intake and risk of celiac disease: long-term follow-up of an at-risk birth cohort. Am. J. Gastroenterol. 114 (8), 1307–1314. 10.14309/ajg.0000000000000255 31082869 PMC6684402

[B72] Meresse B. Malamut G. Cerf-Bensussan N. (2012). Celiac disease: an immunological jigsaw. Immunity 36 (6), 907–919. 10.1016/j.immuni.2012.06.006 22749351

[B73] Moawad M. H. E. Alkhawaldeh I. M. Naswhan A. J. (2023). Efficacy of probiotics supplementation in amelioration of celiac disease symptoms and enhancement of immune system. World J. Clin. Cases 11 (32), 7741–7744. 10.12998/wjcc.v11.i32.7741 38073702 PMC10698417

[B74] Mozafarybazargany M. Khonsari M. Sokoty L. Ejtahed H. S. Qorbani M. (2023). The effects of probiotics on gastrointestinal symptoms and microbiota in patients with celiac disease: a systematic review and meta-analysis on clinical trials. Clin. Exp. Med. 23 (6), 2773–2788. 10.1007/s10238-022-00987-x 36609792

[B75] Oikarinen M. Puustinen L. Lehtonen J. Hakola L. Simell S. Toppari J. (2021). Enterovirus infections are associated with the development of celiac disease in a birth cohort study. Front. Immunol. 11, 604529. 10.3389/fimmu.2020.604529 33603739 PMC7884453

[B76] Olivares M. Benítez-Páez A. de Palma G. Capilla A. Nova E. Castillejo G. (2018). Increased prevalence of pathogenic bacteria in the gut microbiota of infants at risk of developing celiac disease: the PROFICEL study. Gut Microbes 9 (6), 551–558. 10.1080/19490976.2018.1451276 29672211 PMC6287676

[B77] Östensson M. Montén C. Bacelis J. Gudjonsdottir A. H. Adamovic S. Ek J. (2013). A possible mechanism behind autoimmune disorders discovered by genome-wide linkage and association analysis in celiac disease. PLoS One 8 (8), e70174. 10.1371/journal.pone.0070174 23936387 PMC3732286

[B78] Palma G. D. Capilla A. Nova E. Castillejo G. Varea V. Pozo T. (2012). Influence of milk-feeding type and genetic risk of developing coeliac disease on intestinal microbiota of infants: the PROFICEL study. PLoS One 7 (2), e30791. 10.1371/journal.pone.0030791 22319588 PMC3272021

[B79] Paolella G. Sposito S. Romanelli A. M. Caputo I. (2022). Type 2 transglutaminase in coeliac disease: a key player in pathogenesis, diagnosis and therapy. Int. J. Mol. Sci. 23 (14), 7513. 10.3390/ijms23147513 35886862 PMC9318967

[B80] Pinier M. Fuhrmann G. Galipeau H. J. Rivard N. Murray J. A. David C. S. (2012). The copolymer P(HEMA-co-SS) binds gluten and reduces immune response in gluten-sensitized mice and human tissues. Gastroenterology 142 (2), 316–325. 10.1053/j.gastro.2011.10.038 22079593

[B81] Pinto-Sanchez M. I. Silvester J. A. Lebwohl B. Leffler D. A. Anderson R. P. Therrien A. (2021). Society for the Study of Celiac Disease position statement on gaps and opportunities in coeliac disease. Nat. Rev. Gastroenterol. Hepatol. 18 (12), 875–884. 10.1038/s41575-021-00511-8 34526700 PMC8441249

[B82] Plaza-Izurieta L. Fernandez-Jimenez N. Irastorza I. Jauregi-Miguel A. Romero-Garmendia I. Vitoria J. C. (2015). Expression analysis in intestinal mucosa reveals complex relations among genes under the association peaks in celiac disease. Eur. J. Hum. Genet. 23 (8), 1100–1105. 10.1038/ejhg.2014.244 25388004 PMC4795102

[B83] PubMed (2024). Removing celiac disease-related gluten proteins from bread wheat while retaining technological properties: a study with Chinese Spring deletion lines - PubMed. Available at: https://pubmed.ncbi.nlm.nih.gov/19351412/ (Accessed March 18, 2024). 10.1186/1471-2229-9-41 PMC2670835 19351412

[B84] Ramakrishna B. S. Makharia G. K. Chetri K. Dutta S. Mathur P. Ahuja V. (2016). Prevalence of adult celiac disease in India: regional variations and associations. Official J. Am. Coll. Gastroenterology | ACG. 111 (1), 115–123. 10.1038/ajg.2015.398 26729543

[B85] Ricaño-Ponce I. Zhernakova D. V. Deelen P. Luo O. Li X. Isaacs A. (2016). Refined mapping of autoimmune disease associated genetic variants with gene expression suggests an important role for non-coding RNAs. J. Autoimmun. 68, 62–74. 10.1016/j.jaut.2016.01.002 26898941 PMC5391837

[B86] Riddle M. S. Murray J. A. Cash B. D. Pimentel M. Porter C. K. (2013). Pathogen-specific risk of celiac disease following bacterial causes of foodborne illness: a retrospective cohort study. Dig. Dis. Sci. 58 (11), 3242–3245. 10.1007/s10620-013-2733-7 23812827

[B87] Roberts S. E. Morrison-Rees S. Thapar N. Benninga M. A. Borrelli O. Broekaert I. (2021). Systematic review and meta-analysis: the incidence and prevalence of paediatric coeliac disease across Europe. Aliment. Pharmacol. Ther. 54 (2), 109–128. 10.1111/apt.16337 34115894

[B88] Rostami-Nejad M. Razzaghi Z. Esmaeili S. Rezaei-Tavirani S. Akbarzadeh Baghban A. Vafaee R. (2020). Immunological reactions by T cell and regulation of crucial genes in treated celiac disease patients. Gastroenterol. Hepatol. Bed Bench. 13 (2), 155–160. 10.22037/ghfbb.v13i2.1921 32308937 PMC7149810

[B89] Saito S. Ota S. Yamada E. Inoko H. Ota M. (2000). Allele frequencies and haplotypic associations defined by allelic DNA typing at HLA class I and class II loci in the Japanese population. Tissue Antigens 56 (6), 522–529. 10.1034/j.1399-0039.2000.560606.x 11169242

[B90] Schuppan D. Mäki M. Lundin K. E. A. Isola J. Friesing-Sosnik T. Taavela J. (2021). A randomized trial of a transglutaminase 2 inhibitor for celiac disease. N. Engl. J. Med. 385 (1), 35–45. 10.1056/NEJMoa2032441 34192430

[B91] Sciurti M. Fornaroli F. Gaiani F. Bonaguri C. Leandro G. Di Mario F. (2018). Genetic susceptibilty and celiac disease: what role do HLA haplotypes play? Acta Biomed. 89 (9-S), 17–21. 10.23750/abm.v89i9-S.7953 PMC6502200 30561391

[B92] Seiler C. L. Kiflen M. Stefanolo J. P. Bai J. C. Bercik P. Kelly C. P. (2020). Probiotics for celiac disease: a systematic review and meta-analysis of randomized controlled trials. Am. J. Gastroenterol. 115 (10), 1584–1595. 10.14309/ajg.0000000000000749 32740074

[B93] Sellitto M. Bai G. Serena G. Fricke W. F. Sturgeon C. Gajer P. (2012). Proof of concept of microbiome-metabolome analysis and delayed gluten exposure on celiac disease autoimmunity in genetically at-risk infants. PLoS One 7 (3), e33387. 10.1371/journal.pone.0033387 22432018 PMC3303818

[B94] Senapati S. Gutierrez-Achury J. Sood A. Midha V. Szperl A. Romanos J. (2015). Evaluation of European coeliac disease risk variants in a north Indian population. Eur. J. Hum. Genet. 23 (4), 530–535. 10.1038/ejhg.2014.137 25052311 PMC4666579

[B95] Sharma A. Liu X. Hadley D. Hagopian W. Liu E. Chen W. M. (2016). Identification of non-HLA genes associated with celiac disease and country-specific differences in a large, international pediatric cohort. PLoS One 11 (3), e0152476. 10.1371/journal.pone.0152476 27015091 PMC4807782

[B96] Silano M. Agostoni C. Sanz Y. Guandalini S. (2016). Infant feeding and risk of developing celiac disease: a systematic review. BMJ Open 6 (1), e009163. 10.1136/bmjopen-2015-009163 PMC4735130 26810996

[B97] Slifer Z. M. Krishnan B. R. Madan J. Blikslager A. T. (2021). Larazotide acetate: a pharmacological peptide approach to tight junction regulation. Am. J. Physiol. Gastrointest. Liver Physiol. 320 (6), G983–G989. 10.1152/ajpgi.00386.2020 33881350 PMC11735010

[B98] Smyth D. J. Plagnol V. Walker N. M. Cooper J. D. Downes K. Yang J. H. M. (2008). Shared and distinct genetic variants in type 1 diabetes and celiac disease. N. Engl. J. Med. 359 (26), 2767–2777. 10.1056/NEJMoa0807917 19073967 PMC2840835

[B99] Soheilian Khorzoghi M. Rostami-Nejad M. Yadegar A. Dabiri H. Hadadi A. Rodrigo L. (2023). Impact of probiotics on gut microbiota composition and clinical symptoms of coeliac disease patients following gluten-free diet. Contemp. Clin. Trials Commun. 35, 101201. 10.1016/j.conctc.2023.101201 37680267 PMC10480319

[B100] Sollid L. M. Qiao S. W. Anderson R. P. Gianfrani C. Koning F. (2012). Nomenclature and listing of celiac disease relevant gluten T-cell epitopes restricted by HLA-DQ molecules. Immunogenetics 64 (6), 455–460. 10.1007/s00251-012-0599-z 22322673 PMC3349865

[B101] Stene L. C. Honeyman M. C. Hoffenberg E. J. Haas J. E. Sokol R. J. Emery L. (2006). Rotavirus infection frequency and risk of celiac disease autoimmunity in early childhood: a longitudinal study. Am. J. Gastroenterol. 101 (10), 2333–2340. 10.1111/j.1572-0241.2006.00741.x 17032199

[B102] Szajewska H. Shamir R. Chmielewska A. Pieścik-Lech M. Auricchio R. Ivarsson A. (2015). Systematic review with meta-analysis: early infant feeding and coeliac disease--update 2015. Aliment. Pharmacol. Ther. 41 (11), 1038–1054. 10.1111/apt.13163 25819114

[B103] Tapia G. Chudá K. Kahrs C. R. Stene L. C. Kramna L. Mårild K. (2021). Parechovirus infection in early childhood and association with subsequent celiac disease. Official J. Am. Coll. Gastroenterology | ACG. 116 (4), 788–795. 10.14309/ajg.0000000000001003 33982949

[B104] Tosi R. Vismara D. Tanigaki N. Ferrara G. B. Cicimarra F. Buffolano W. (1983). Evidence that celiac disease is primarily associated with a DC locus allelic specificity. Clin. Immunol. Immunopathol. 28 (3), 395–404. 10.1016/0090-1229(83)90106-X 6192959

[B105] Trynka G. Hunt K. A. Bockett N. A. Romanos J. Mistry V. Szperl A. (2011). Dense genotyping identifies and localizes multiple common and rare variant association signals in celiac disease. Nat. Genet. 43 (12), 1193–1201. 10.1038/ng.998 22057235 PMC3242065

[B106] Trynka G. Zhernakova A. Romanos J. Franke L. Hunt K. A. Turner G. (2009). Coeliac disease-associated risk variants in TNFAIP3 and REL implicate altered NF-kappaB signalling. Gut 58 (8), 1078–1083. 10.1136/gut.2008.169052 19240061

[B107] Valitutti F. Cucchiara S. Fasano A. (2019). Celiac disease and the microbiome. Nutrients 11 (10), 2403. 10.3390/nu11102403 31597349 PMC6835875

[B108] van Heel D. A. Franke L. Hunt K. A. Gwilliam R. Zhernakova A. Inouye M. (2007). A genome-wide association study for celiac disease identifies risk variants in the region harboring IL2 and IL21. Nat. Genet. 39 (7), 827–829. 10.1038/ng2058 17558408 PMC2274985

[B109] Varma S. Krishnareddy S. (2022). Novel drug therapeutics in celiac disease: a pipeline review. Drugs 82 (15), 1515–1526. 10.1007/s40265-022-01784-2 36251239

[B110] Volta U. Villanacci V. (2011). Celiac disease: diagnostic criteria in progress. Cell Mol. Immunol. 8 (2), 96–102. 10.1038/cmi.2010.64 21278763 PMC4003134

[B111] Vriezinga S. L. Auricchio R. Bravi E. Castillejo G. Chmielewska A. Crespo Escobar P. (2014). Randomized feeding intervention in infants at high risk for celiac disease. N. Engl. J. Med. 371 (14), 1304–1315. 10.1056/NEJMoa1404172 25271603

[B112] Wijarnpreecha K. Lou S. Panjawatanan P. Cheungpasitporn W. Pungpapong S. Lukens F. J. (2018). Cigarette smoking and risk of celiac disease: a systematic review and meta-analysis. United Eur. Gastroenterol. J. 6 (9), 1285–1293. 10.1177/2050640618786790 PMC6206527 30386601

[B113] Wijmenga C. Gutierrez-Achury J. (2014). Celiac disease genetics: past, present and future challenges. J. Pediatr. Gastroenterol. Nutr. 59 (Suppl. 1), S4–S7. 10.1097/01.mpg.0000450392.23156.10 24979196

[B114] Yuan J. Zhou C. Gao J. Li J. Yu F. Lu J. (2017). Prevalence of celiac disease autoimmunity among adolescents and Young adults in China. Clin. Gastroenterology Hepatology 15 (10), 1572–1579. 10.1016/j.cgh.2017.04.025 28433781

[B115] Zhernakova A. Elbers C. C. Ferwerda B. Romanos J. Trynka G. Dubois P. C. (2010). Evolutionary and functional analysis of celiac risk loci reveals SH2B3 as a protective factor against bacterial infection. Am. J. Hum. Genet. 86 (6), 970–977. 10.1016/j.ajhg.2010.05.004 20560212 PMC3032060

[B116] Zhernakova A. Stahl E. A. Trynka G. Raychaudhuri S. Festen E. A. Franke L. (2011). Meta-analysis of genome-wide association studies in celiac disease and rheumatoid arthritis identifies fourteen non-HLA shared loci. PLoS Genet. 7 (2), e1002004. 10.1371/journal.pgen.1002004 21383967 PMC3044685

