# Supplementary material for: From an understanding of etiopathogenesis to novel therapies—what is new in the treatment of celiac disease?
Source: Front Pharmacol. 2024 Apr 18;15:1378172. doi: 10.3389/fphar.2024.1378172 (PMC11063403; doi:10.3389/fphar.2024.1378172)
Supplement: Supplementary file 1 [file Table1.docx]

**Table 1.** Genetic risk variants associated with celiac disease. Based on R. Dieli-Crimi et al. Journal of Autoimmunity 64 (2015) 26e41, [doi.org/10.1016/j.jaut.2015.07.003](https://doi.org/10.1016/j.jaut.2015.07.003)

| **Genetic determinants** | **Localization** | **Immunological function** | **References**  **(confirming role in Celiac)** |
| --- | --- | --- | --- |
| *NFIA* | 1p31 | Differentiation of myeloid lineages. Central nervous system development | [1] |
| *PARK7*  *TNFRSF9* | 1p36.23 | *PARK7*: role in cell protection against oxidative stress and cell death  *TNFRSF9:* contributes to the clonal expansion, survival, and development of T cells. It can also induce proliferation in peripheral monocytes | [2] |
| *TNFRSF14*  *MMEL1* | 1p36.32 | TNFRSF14: signal transduction pathways influencing immune response;  MMEL1: phosphate metabolism  and homeostasis | [2] |
| CD247 | 1q24.3 | Plays an important role in coupling antigen recognition to several intracellular signal-transduction pathways. Low expression of the antigen results in impaired immune response. | [3] |
| *FASLG, TNFSF18* | 1q24.3 | FASLG: essential for immune system regulation, including activation-induced cell death (AICD) of T cells and cytotoxic T lymphocyte-induced cell death  TNFSF18: modulate T lymphocyte survival, important for interaction between T lymphocytes and endothelial cells | [4,5] |
| *NCF2* | 1q25 | Oxidative stress | [6] |
| RGS1 | 1q31.2 | B-cell activation and proliferation. Cell signaling and migration | [7] |
| *DUSP10* | 1q41 | Innate immunity and oxidative stress | [8] |
| *PLEK*  *FBXO48* | 2p13 | PLEK: cytoskeletal reorganization | [9] |
| *REL*  *AHSA2*  *PUS10* | 2p16.1 | REL: NF-kB signaling pathway; AHSA2: acts as co-chaperone of Hsp90 (heat shock protein 90) PUS10: posttranscriptional nucleotide modification | [10] |
| IL1RL1  IL1RL2  IL18R1  IL18RAP  SLC9A4 | 2q12.1 | IL1RL1, IL18R1, IL18RAP:  involved in inducing cell-mediated immunity. pro-inflammatory role;  SLC9A4:  involved in potassium ion transmembrane transport; regulation of intracellular pH; | [11,12] |
| *ITGA4*  *UBE2E3* | 2q31.3 | ITGA4: cell adhesion, migration, and activation of immune cells; UBE2E3: ubiquitination | [13] |
| *STAT4* | 2q32 | key role in the differentiation of T-helper 1 cells and the production of interferon-gamma | [5] |
| *CTLA4*  *ICOS*  *CD28* | 2q33.2 | T and B cell activation and proliferation (CD28, ICOS: stimulation; CTLA4: inhibition) | [14] |
| *ARHGAP31* | 3p13 | involved in protein trafficking and cell growth | [15] |
| *FRMD4B* | 3p14.1 | May function as a scaffolding protein that regulates epithelial cell polarity | [16] |
| *CCR1,*  *CCR2,*  *CCR3,*  *CCR5,*  *CCR9,*  *CCRL2,* | 3p21.31 | Cell differentiation, recruitment, and signaling, protection from inflammatory response | [4] |
| *CCR4, GLB1* | 3p22.3 | CCR4: regulate cell trafficking of various types of leukocytes- play fundamental roles in the development, homeostasis, and function of the immune system; GLB1: hydrolysis of glycoconjugates | [17,18] |
| *IL12A, SCHIP1,* | 3p25.33 | IL12A:  T-cell-independent induction of interferon (IFN)-gamma, differentiation of Th1 and Th2 cells; SCHIP1: tumor suppression | [7] |
| *CD80, POGLUT1* | 3q13.33 | *CD80:* activated protein induces T-cell proliferation and cytokine production  POGLUT1: helps with protein processing and transport | [3,4] |
| *LPP* | 3q28 | involved in signal transduction from cell adhesion sites to the nucleus | [19] |
| KIAA1109, ADAD1,  IL2,  IL21 | 4q27 | IL2, IL21: B- and T-cell activation and proliferation | [20] |
| HLA-DQA1, HLA-DQB1 | 6p21.32 | Gluten recognition and CD4^+^ T cell presentation | [21,22] |
| *HLA-B* | 6p21 | Peptide recognition and CD8 T cell presentation | [23] |
| *HLA-DPB1* | 6p21 | Peptide recognition and CD4 T cell presentation | [24] |
| *HLA-F* | 6p21 | play a role in immune surveillance, immune tolerance, and inflammation, peptide recognition, and CD8 T cell presentation | [25] |
| *IRF4* | 6p25.3 | Lymphocyte-specific regulator of TLR signaling. Involved in innate and adaptive immunity | [4] |
| *BACH2 , MAP3K7* | 6q15 | Regulation of humoral and cellular immunity | [4] |
| *PTPRK, THEMIS* | 6q22.33 | PTPRK: barrier function, CD4 T cell development; THEMIS: thymic differentiation of CD4þ T cells | [26] |
| *OLIG3, TNFAIP3* | 6q23.3 | OLIG3: neuronal development; TNFAIP3: NF-kB signaling pathway and TNF-mediated apoptosis | [10] |
| *ELMO1* | 7q14.1 | Involved in phagocytosis and cell migration | [4] |
| *PVT1* | 8q24 | Participation in recurrent translocations |  |
| *PFKFB3, PRKCQ* | 10p15 | PFKFB3: oxidative stress regulation; PRKCQ: T cell activation and NF-kB signaling pathway | [27] |
| *ZMIZ1* | 10q22.3 | Regulation of transcription factor activation | [4] |
| *POU2AF1,*  *POU2AF3* | 11q23 | POU2AF1: B-cell development and function | [28] |
| *TREH, DDX6* | 11q23 | TREH: hydrolysis of trehalose, stress-response protein; DDX6: mRNA degradation and suppression of  protein translation  HCFC1: control of the cell cycle and transcriptional regulation; IRAK1: innate immunity | [1] |
| *ETS1* | 11q24.3 | Differentiation, survival, and proliferation of lymphoid cells | [4] |
| *SH2B3*  *ATXN2* | 12q24.12 | SH2B3: innate immune response and T cell signaling; ATXN2: stabilization of mRNAs | [29] |
| *ZFP36L1* | 14q24.1 | Regulation of the response to growth factors, wound healing | [4] |
| *CLK3,*  *CSK,*  *ULK3* | 15q24 | CLK3: regulation of alternative splicing; CSK: (relevant to T cell receptor (TCR) signaling regulation of cell growth, differentiation, migration, and immune response; ULK3: relevant for autophagy | [30–32] |
| *CIITA,*  *SOCS1, CLEC16A* | 16p13.13 | CIITA: regulation of HLA class II genes transcription | [4] |
| *SOCS1,*  *PRM1,*  *PRM2* | 16q13 | SOCS1: regulation of cytokine signaling; PRM1 and PRM2: germ cell development | [33–35] |
| *PTPN2* | 18p11.21 | T-cell differentiation and activation | [36] |
| *ZNF335* | 20q13 | Regulation of gene expression, chromatin remodeling | [23] |
| *UBASH3A* | 21q22 | T-cell signaling and T-cell apoptosis | [37] |
| *ICOSLG* | 21q22.3 | T and B cell activation and proliferation | [4] |
| *UBE2L3*  *YDJC* | 22q11.21 | UBE2L3:NF-kB signaling pathway and ubiquitination | [4] |
| *TLR7, TLR8* | Xp22.2 | *TLR7 and TLR8*: role in pathogen recognition and activation of innate immunity. | [4] |
| *HCFC1, TMEM187,*  *IRAK1* | Xq28 | HCFC1: Involved in the control of the cell cycle and transcriptional regulation; IRAK1 plays a critical role in initiating innate immune response | [1] |

[1] Coleman C, Quinn EM, Ryan AW, Conroy J, Trimble V, Mahmud N, et al. Common polygenic variation in coeliac disease and confirmation of ZNF335 and NIFA as disease susceptibility loci. Eur J Hum Genet 2016;24:291–7. https://doi.org/10.1038/ejhg.2015.87.

[2] Dubois PCA, Trynka G, Franke L, Hunt KA, Romanos J, Curtotti A, et al. Multiple common variants for celiac disease influencing immune gene expression. Nat Genet 2010;42:295–302. https://doi.org/10.1038/ng.543.

[3] Dieli-Crimi R, Cénit MC, Núñez C. The genetics of celiac disease: A comprehensive review of clinical implications. Journal of Autoimmunity 2015;64:26–41. https://doi.org/10.1016/j.jaut.2015.07.003.

[4] Abadie V, Sollid LM, Barreiro LB, Jabri B. Integration of genetic and immunological insights into a model of celiac disease pathogenesis. Annu Rev Immunol 2011;29:493–525. https://doi.org/10.1146/annurev-immunol-040210-092915.

[5] Trynka G, Hunt KA, Bockett NA, Romanos J, Mistry V, Szperl A, et al. Dense genotyping identifies and localizes multiple common and rare variant association signals in celiac disease. Nat Genet 2011;43:1193–201. https://doi.org/10.1038/ng.998.

[6] Hunt KA, Mistry V, Bockett NA, Ahmad T, Ban M, Barker JN, et al. Negligible impact of rare autoimmune-locus coding-region variants on missing heritability. Nature 2013;498:232–5. https://doi.org/10.1038/nature12170.

[7] Guo C-C, Wang M, Cao F-D, Huang W-H, Xiao D, Ye X-G, et al. Meta-Analysis on Associations of RGS1 and IL12A Polymorphisms with Celiac Disease Risk. Int J Mol Sci 2016;17:457. https://doi.org/10.3390/ijms17040457.

[8] Östensson M, Montén C, Bacelis J, Gudjonsdottir AH, Adamovic S, Ek J, et al. A possible mechanism behind autoimmune disorders discovered by genome-wide linkage and association analysis in celiac disease. PLoS One 2013;8:e70174. https://doi.org/10.1371/journal.pone.0070174.

[9] Sharma A, Liu X, Hadley D, Hagopian W, Liu E, Chen W-M, et al. Identification of Non-HLA Genes Associated with Celiac Disease and Country-Specific Differences in a Large, International Pediatric Cohort. PLoS One 2016;11:e0152476. https://doi.org/10.1371/journal.pone.0152476.

[10] Trynka G, Zhernakova A, Romanos J, Franke L, Hunt KA, Turner G, et al. Coeliac disease-associated risk variants in TNFAIP3 and REL implicate altered NF-kappaB signalling. Gut 2009;58:1078–83. https://doi.org/10.1136/gut.2008.169052.

[11] Akhabir L, Sandford A. Genetics of Interleukin 1 Receptor-Like 1 in Immune and Inflammatory Diseases. Curr Genomics 2010;11:591–606. https://doi.org/10.2174/138920210793360907.

[12] Balasopoulou A, Stanković B, Panagiotara A, Nikčevic G, Peters BA, John A, et al. Novel genetic risk variants for pediatric celiac disease. Hum Genomics 2016;10:34. https://doi.org/10.1186/s40246-016-0091-1.

[13] Garner CP, Murray JA, Ding YC, Tien Z, van Heel DA, Neuhausen SL. Replication of celiac disease UK genome-wide association study results in a US population. Hum Mol Genet 2009;18:4219–25. https://doi.org/10.1093/hmg/ddp364.

[14] Smyth DJ, Plagnol V, Walker NM, Cooper JD, Downes K, Yang JHM, et al. Shared and distinct genetic variants in type 1 diabetes and celiac disease. N Engl J Med 2008;359:2767–77. https://doi.org/10.1056/NEJMoa0807917.

[15] Discepolo V, Lania G, Ten Eikelder MLG, Nanayakkara M, Sepe L, Tufano R, et al. Pediatric Celiac Disease Patients Show Alterations of Dendritic Cell Shape and Actin Rearrangement. International Journal of Molecular Sciences 2021;22:2708. https://doi.org/10.3390/ijms22052708.

[16] Garner C, Ahn R, Ding YC, Steele L, Stoven S, Green PH, et al. Genome-Wide Association Study of Celiac Disease in North America Confirms FRMD4B as New Celiac Locus. PLoS One 2014;9:e101428. https://doi.org/10.1371/journal.pone.0101428.

[17] Plaza-Izurieta L, Fernandez-Jimenez N, Irastorza I, Jauregi-Miguel A, Romero-Garmendia I, Vitoria JC, et al. Expression analysis in intestinal mucosa reveals complex relations among genes under the association peaks in celiac disease. Eur J Hum Genet 2015;23:1100–5. https://doi.org/10.1038/ejhg.2014.244.

[18] Gell G, Kovács K, Veres G, Korponay-Szabó IR, Juhász A. Characterization of globulin storage proteins of a low prolamin cereal species in relation to celiac disease. Sci Rep 2017;7:39876. https://doi.org/10.1038/srep39876.

[19] Huang S-Q, Zhang N, Zhou Z-X, Huang C-C, Zeng C-L, Xiao D, et al. Association of LPP and TAGAP Polymorphisms with Celiac Disease Risk: A Meta-Analysis. Int J Environ Res Public Health 2017;14:171. https://doi.org/10.3390/ijerph14020171.

[20] van Heel DA, Franke L, Hunt KA, Gwilliam R, Zhernakova A, Inouye M, et al. A genome-wide association study for celiac disease identifies risk variants in the region harboring IL2 and IL21. Nat Genet 2007;39:827–9. https://doi.org/10.1038/ng2058.

[21] Tosi R, Vismara D, Tanigaki N, Ferrara GB, Cicimarra F, Buffolano W, et al. Evidence that celiac disease is primarily associated with a DC locus allelic specificity. Clinical Immunology and Immunopathology 1983;28:395–404. https://doi.org/10.1016/0090-1229(83)90106-X.

[22] Sollid LM, Qiao S-W, Anderson RP, Gianfrani C, Koning F. Nomenclature and listing of celiac disease relevant gluten T-cell epitopes restricted by HLA-DQ molecules. Immunogenetics 2012;64:455–60. https://doi.org/10.1007/s00251-012-0599-z.

[23] Gutierrez-Achury J, Zhernakova A, Pulit SL, Trynka G, Hunt KA, Romanos J, et al. Fine mapping in the MHC region accounts for 18% additional genetic risk for celiac disease. Nat Genet 2015;47:577–8. https://doi.org/10.1038/ng.3268.

[24] Klobuch S, Lim JJ, van Balen P, Kester MGD, de Klerk W, de Ru AH, et al. Human T cells recognize HLA-DP–bound peptides in two orientations. Proceedings of the National Academy of Sciences 2022;119:e2214331119. https://doi.org/10.1073/pnas.2214331119.

[25] Sciurti M, Fornaroli F, Gaiani F, Bonaguri C, Leandro G, Di Mario F, et al. Genetic susceptibilty and celiac disease: what role do HLA haplotypes play? Acta Biomed 2018;89:17–21. https://doi.org/10.23750/abm.v89i9-S.7953.

[26] Bondar C, Plaza-Izurieta L, Fernandez-Jimenez N, Irastorza I, Withoff S, Wijmenga C, et al. THEMIS and PTPRK in celiac intestinal mucosa: coexpression in disease and after in vitro gliadin challenge. Eur J Hum Genet 2014;22:358–62. https://doi.org/10.1038/ejhg.2013.136.

[27] Senapati S, Gutierrez-Achury J, Sood A, Midha V, Szperl A, Romanos J, et al. Evaluation of European coeliac disease risk variants in a north Indian population. Eur J Hum Genet 2015;23:530–5. https://doi.org/10.1038/ejhg.2014.137.

[28] Kumar V, Gutierrez-Achury J, Kanduri K, Almeida R, Hrdlickova B, Zhernakova DV, et al. Systematic annotation of celiac disease loci refines pathological pathways and suggests a genetic explanation for increased interferon-gamma levels. Human Molecular Genetics 2015;24:397–409. https://doi.org/10.1093/hmg/ddu453.

[29] Zhernakova A, Elbers CC, Ferwerda B, Romanos J, Trynka G, Dubois PC, et al. Evolutionary and Functional Analysis of Celiac Risk Loci Reveals SH2B3 as a Protective Factor against Bacterial Infection. Am J Hum Genet 2010;86:970–7. https://doi.org/10.1016/j.ajhg.2010.05.004.

[30] G T, Ka H, Na B, J R, V M, A S, et al. Dense genotyping identifies and localizes multiple common and rare variant association signals in celiac disease. Nat Genet 2011;43:1193–201. https://doi.org/10.1038/ng.998.

[31] Bakker OB, Ramírez-Sánchez AD, Borek ZA, de Klein N, Li Y, Modderman R, et al. Potential impact of celiac disease genetic risk factors on T cell receptor signaling in gluten-specific CD4+ T cells. Sci Rep 2021;11:9252. https://doi.org/10.1038/s41598-021-86612-5.

[32] Ricaño-Ponce I, Zhernakova DV, Deelen P, Luo O, Li X, Isaacs A, et al. Refined mapping of autoimmune disease associated genetic variants with gene expression suggests an important role for non-coding RNAs. J Autoimmun 2016;68:62–74. https://doi.org/10.1016/j.jaut.2016.01.002.

[33] Hadjadj J, Castro CN, Tusseau M, Stolzenberg M-C, Mazerolles F, Aladjidi N, et al. Early-onset autoimmunity associated with SOCS1 haploinsufficiency. Nat Commun 2020;11:5341. https://doi.org/10.1038/s41467-020-18925-4.

[34] Rostami-Nejad M, Razzaghi Z, Esmaeili S, Rezaei-Tavirani S, Akbarzadeh Baghban A, Vafaee R. Immunological reactions by T cell and regulation of crucial genes in treated celiac disease patients. Gastroenterol Hepatol Bed Bench 2020;13:155–60.

[35] Meresse B, Malamut G, Cerf-Bensussan N. Celiac disease: an immunological jigsaw. Immunity 2012;36:907–19. https://doi.org/10.1016/j.immuni.2012.06.006.

[36] Festen EAM, Goyette P, Green T, Boucher G, Beauchamp C, Trynka G, et al. A meta-analysis of genome-wide association scans identifies IL18RAP, PTPN2, TAGAP, and PUS10 as shared risk loci for Crohn’s disease and celiac disease. PLoS Genet 2011;7:e1001283. https://doi.org/10.1371/journal.pgen.1001283.

[37] Zhernakova A, Stahl EA, Trynka G, Raychaudhuri S, Festen EA, Franke L, et al. Meta-analysis of genome-wide association studies in celiac disease and rheumatoid arthritis identifies fourteen non-HLA shared loci. PLoS Genet 2011;7:e1002004. https://doi.org/10.1371/journal.pgen.1002004.
